# A family carrying a homozygous *LACC1* truncated mutation expands the clinical phenotype of this disease beyond systemic-onset juvenile idiopathic arthritis

**DOI:** 10.1186/1546-0096-13-S1-O76

**Published:** 2015-09-28

**Authors:** JI Arostegui, R Rabionet, A Remesal, A Mensa-Vilaro, S Murias, R Alcobendas, E Gonzalez-Roca, O Dreschsel, E Ruiz-Ortiz, A Puig, D Comas, S Ossowski, J Yagüe, X Estivill, R Merino

**Affiliations:** 1 Hospital Clinic, Immunology, Barcelona, Spain; 2 Center for Genomic Regulation, Barcelona, Spain; 3 Hospital La Paz, Pediatric Rheumatology, Madrid, Spain; 4 Universitat Pompeu Fabra, Barcelona, Spain

## Introduction

We identified a consanguineous Moroccan family with three affected siblings diagnosed with rheumatoid factor-negative polyarticular juvenile idiopathic arthritis. They all suffered from an early-onset (2-4 years-old) chronic and symmetric polyarthritis affecting both large and small joints. The joint involvement was markedly erosive in two siblings, with the older sister requiring hip prosthetic replacement at the age of 18 years. None of the patients had fever, skin rash, uveitis or other extra-articular manifestations. Laboratory analyses revealed leukocytosis, thrombocytosis, severe anaemia, marked increase of inflammatory markers and negative results for rheumatoid factor, anti-nuclear antibodies and HLA-B27.

## Objective

We suspected a genetic cause for the disease in this family on the basis of the atypical phenotype, the presence of consanguinity and the recurrence of the disease following a recessive mode of inheritance. The aim of this study was to identify the causal genetic defect underlying this family by means the use of novel genetic technologies.

## Patients and methods

We performed genome-wide SNP analyses and whole-exome sequencing in both affected and unaffected siblings to identify those homozygosity regions that were exclusively shared by patients, and the candidate causal gene variants inside these regions, respectively.

## Results

Four homozygosity regions were identified in chromosomes 3, 6 (n: 2) and 13, containing over 330 genes. Whole-exome sequencing identified three potential candidate variants within these regions, located in the *TATDN2, FARS2*, and *LACC1* genes, respectively. Bioinformatics and genetic studies performed in a group of healthy Moroccan individuals (n: 352) finally supported the frameshift c.128_129delGT variant in the *LACC1* gene, leading to a truncated protein (p.Cys43Tyrfs*6), as the most probable causal gene defect

## Conclusions

Our findings show homozygous *LACC1* mutations as the genetic defect underlying a severe inflammatory joint disease with a recessive mode of inheritance. These evidences expand the phenotype of this rare genetic disorder to other forms of juvenile idiopathic arthritis in addition to the previously described systemic-onset juvenile idiopathic arthritis [1].
